# Structural model of resilience based on parental support: the mediating role of hope and active coping

**DOI:** 10.1186/s12888-023-04678-z

**Published:** 2023-04-17

**Authors:** Hadis Cheraghian, Khadije Moradian, Tahere Nouri

**Affiliations:** 1 Mohaghegh Ardabili University, Ardabil, Iran; 2 Lorestan University, Lorestan, Iran; 3 Allame Tabatabaie University, Tehran, Iran

**Keywords:** Resilience, Hope, Cope, Paternal supports

## Abstract

Resilience is an essential trait in the academic and non-academic lives of students that has been associated with positive psychological and non-psychological outcomes. Given the importance and role of resilience, the main goal of this study was to create a structural model of resilience based on parental support and emphasizing the mediating role of hope and coping. The research design was correlational in nature and used structural equation modeling (SEM). The population of this study included high school students in Tehran city in 2019–2020 school year, and the sample consisted of 560 students selected by cluster sampling method. By applying Spearman correlation analysis and structural equation modeling, a significant relationship between hope, coping, resilience, and parental support was found. Both the SEM measurement and structural models provided a good fit. The significant findings of the present study include the direct and significant effect of parental support on resilience. The indirect and significant effect of parental support on resilience through the mediating role of hope and coping was also confirmed. Overall, the results of this study show that the effect of parental support on resilience can be improved by promoting hope and coping styles.

## Introduction

The need to be close to parents when necessary and the need of parents to respond to their children's need is biologically based on the survival of the species. Adolescents continue to use parents as a safe base for exploration and temporarily return to the parents' safe haven to help them, especially in times of distress, illness, anxiety, or stress [1]. Parents are one of the most important contexts in which children accomplish their developmental tasks. Specifically parental support based on warmth or the expression of love, affection, interest, and contentment toward children can form the basis for improving the sense of belonging and well-being and vitality [2], and reducing depression [3].

Thus, parents play a key role in raising and supporting their children through all stages of development. They pave the way for adaptations to emerge so that children can successfully overcome many challenges [4]. In addition, parental support can protect the child from stressors that are detrimental to their physical and mental health and provide the foundation for social appropriateness and effective learning [5].

Masten [6] discussed that the parent relationship to resilience has been confirmed by every review of the literature since resilience research began. Resilience is the successful coping with injuries, stressors, and difficult situations. Resilience is an interactive process among three factors: children's intrinsic values, the family's supportive role, and the supportive community [7]. The effects of parental support on resilience have also been the focus of researchers and theorists for decades [4]. These effects may be due to the fact that parents play many roles in a child's growth, learning, and well-being [8]. In this context, Doty, Davis, & Arditti [9] propose a model of resilience in which family support is a facilitator of resilience. Also those who exhibit higher levels of resilience tend to actively cope with stressful situations [10, 11].

Higher levels of resilience were related to more effective coping strategies, including better problem solving and less avoidant coping [12]. Believes that parenting supportive and talking about dreams and goals create a sense in a person that they are in control of their environment. The presence of a supportive environment makes it possible to pursue goals, and in this way promotes hope and Active coping. In addition to emphasizing the effects of parental support on resilience, some researchers believe that a number of variables may play a mediating role in this effect. Active coping and hope are among the variables that have a particular impact on parental support in the first stage and influence and promote resilience in the second stage [4, 13–17].

### Active coping

Coping refers to cognitive and behavioral strategies for coping with stressful events or negative psychological and physical outcomes [18]. However, all existing definitions of coping emphasize the reaction process. They assume that the response process is controlled and require efforts to ensure that all individual responses, behaviors, and thoughts are purposeful, conscious, and intentional. Coping styles are considered stable strategies for managing or tolerating external and internal pressures or stressors. Some people actively cope with stress, while others cope passively. Active coping is one of the structures for which there is no general and clear agreement on its definition [19]. Active coping strategies are either behaviors or psychological responses aimed at changing the nature of stressors or one's psyche, whereas passive coping strategies result in people engaging in activities (such as alcohol consumption) or mental states (such as withdrawal) that prevent them from dealing directly with stressful events. Coping behaviors help explain why the same stressors can lead to burnout in some individuals but not in others [20]. In this study, the definition of Compas et al. [21] was used to define coping. They consider coping to be a voluntary and conscious effort to regulate emotions, cognition, behavior, physiology, and environment in response to stressful events and environments [21]. This definition is based on the control-driven model of coping. In this model, coping includes primary coping (attempting to directly influence the source of stress by using solutions such as problem solving and emotional expressions), secondary coping (attempting to adapt to the stress barrier through methods such as cognitive acceptance and reconstruction), and coping through ignorance (Attempt to deflect the source of stress through methods such as avoidance and denial) [22]. People use coping strategies and parent support to strengthen their adaptive capacity, and resilient people rely on favorable dispositional attitudes and behaviors, such as an internal locus of control, to cope with stress [23]. In addition to active coping, hope also appears to play a mediating role in the relationship between parental support and resilience [16]. Our conceptual framework highlights the importance of adaptive coping by adolescent.

### Hope

Hope in difficult situations, but also for uncertainty and scarcity, is an important factor for individual adaptation [24]. The concept of hope is challenging for researchers because it is complex and intangible. However, most researchers are aware that hope is an adaptive force that helps individuals achieve their goals in difficult situations. In fact, this structure plays an important role in reducing depression, anxiety, confusion, and symptoms associated with various diseases [25]. According to Snyder's [12] theory of hope, it can be concluded that hope is a learned thought pattern or a set of beliefs and thoughts that help an individual to achieve his goals according to his abilities. In this regard, many researchers assume that there is a direct relationship between hope and resilience [26, 27].

Overall adolescent build their resilience, hope, optimism, emotional regulation, and wisdom on their successful experiences in coping with difficult life events [28]. These characteristics of resilience require researchers to examine resilience development from a multilevel perspective. The conceptual framework of this study is based on the resilient systems model and assumes that individual development is an interactive process that emanates from internal and external factors [29]. In general, concerns about disruption due to technological advances, economic and educational problems, individual and social impairments, and family problems have led researchers to pay particular attention to resilience as an attachment structure [30].

Our proposed framework suggests that resilience-promoting factors may operate in an interactive and accumulative manner and Resilience refers to characteristics or mental processes by which people can recover from stressors and refrain from physical or emotional disturbances. In general, concerns about disruption due to technological advances, economic and educational problems, individual and social impairments, and family problems have led researchers to pay particular attention to resilience as an attachment structure [30]. The greater the stress, the greater the resources needed to promote psychological resilience [8]. Although various researches have been conducted on the structure of resilience, it remains difficult to create a model that integrates the individual research and shows how resilience can be promoted in a coherent formation. Thus, the role of parental support in resilience motivated the researcher to investigate and explore this important topic. Although extensive research has examined the relationship between resilience and coping styles, the relationship between resilience and coping in patients in particular has been studied [31]. Also, the study of these variables in research has not been conducted in an integrated manner and using a model. In addition, the research gap and lack of research background in the field of resilience model and active coping is a major gap in research. More importantly, there are few relevant studies among students in Iran. Considering the importance of resilience and possible effective factors, this study aims to develop a structural model of resilience that considers the role of parental support, active coping, and hope among students in Iran. Thus, the main question of the study is: Is there a relationship between resilience and parental support, active coping, and hope to formulate a structural model of resilience?

The one Hypothesis: Parental support has a direct influence on resilience.

The Second hypothesis: active coping and hope mediate the relationship between parental support and resilience.

## Method

### Participants

The target population was high school students in the city of Tehran, Iran, in the academic year 2019–2020. Participants were selected using the cluster sampling method. First, we coordinated with the Ministry of Education in the city of Tehran and then generated a list of high schools. Public, private, and special schools (with entrance examination) were classified in each period of high schools. Based on the number of students in each category, the number of schools was determined proportionally to their size. Cluster sampling was used to recruit participants from all four regions of Tehran: North, South, East, and West. The number of students recruited from each region was determined according to their proportion in the total number of students in the regions. Stratified random sampling was used to select the classes in each school to complete the questionnaire. Criteria for participation in the study included belonging to a student group, being between 14 and 17 years of age, and willingness to cooperate. The only criterion for exclusion from the study was failure to answer 25% of the questions in the questionnaire.

According to Kline [32], it was suggested to include 5 to 20 observations for each parameter. In this study, there are 17 parameters, and the number of 560 samples seems appropriate.

### Design

The present study is correlational in the form of structural equation models (SEM). The SEM analysis is a combination of confirmatory factor analysis and multiple regression techniques. We introduced two mediator variables, hope and active coping to the model, and analyzed the indirect and direct relations between the variables. We calculated the correlation of the variables. The strategy of the present study is a quantitative and descriptive analysis that describes the distribution of the data of each of the independent and dependent variables through appropriate statistical indicators.

### Measures

The items of the questionnaire were combined based on different studies, and testing their validity and reliability. In structural models, the complexity of the model increases when the number of items is very large and all of them are considered as observed variables, therefore, a researcher-made questionnaire was used. This leads to problems such as the inappropriate adjustment of indicators, the challenge of sample size and the significance of parameter estimation. In this situation, questionnaires with a smaller number of items are preferred [33]. In this regard, Floyd, & Wideman [34] believe that if there are 5 to 8 items for each variable in the structural model, the model is unlikely to fit optimally, so such models are generally not approved. Table 1 shows the factors, items, sources, and validity of each factor. All questionnaires were designed based on the Likert scale. Demographic and socioeconomic factors were collected about the participants: Father's and mother's occupation and education, Number of children, age, gender, field of study and Academic year.

**Table 1:** Factors, items, sources and validity (reliability) of each factor

Factors	Sub scale	Source	Credit
**Parental support**	Intimate relationships with parents	Brown [35]; Benson, & Johnson [36]; Ford et al.[37]	**0.82**
	Parental care		
	Love talking to parents		
	Expressing parental interest		
**Active coping**	Talking to relatives about concerns	Boujut, Bruchon-Schweitzer, & Dombrowski [38]	**0.76**
	Consult others		
	Ask for help to overcome the problem		
	Write down problems or talk about them		
**Hope**	Pursue energetic goals	Snyder et al., [39]	**0.58**
	Considering different solutions		
	Find solutions when many people are frustrated		
	Feeling too tired		
**Resilience**	Having a sense of control over life Having a sense of purpose Do not despair in the face of frustrating things It is expedient to consider any good or bad event	Conner, & Davidson, [40]	**0.66**

**Fig. 1:**
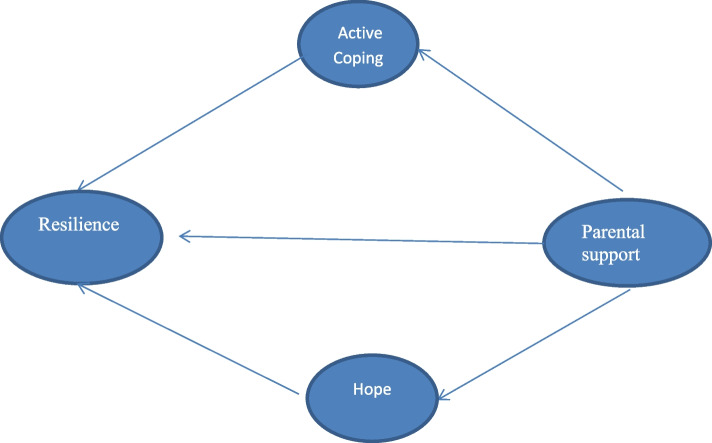
Proposed conceptual model of research

From the Cronbach's alpha presented in Table 1, it can be concluded that all factors of the questionnaire have good reliability. To check the validity of this questionnaire, content validity was used in the first phase, and after confirming this validity by education experts and university professors, construct validity of this questionnaire was investigated by confirmatory factor analysis. Figure 1 shows the standardized path coefficients, error amount, chi-square and root mean square error (RMSEA) index for the factors present in the study (measurement model).

Root mean square error (RMSEA) of less than 0.08 indicates the optimal fit of the model. Thus, as shown in Fig. 1, the research measurement model is well fitted. In terms of the significance of the chi-squared statistic (X2) shown in Fig. 1, it can also be noted that this statistic is sensitive to a large sample size, meaning that the chi-squared test is almost always significant when the sample size is large. [41]. Various researchers use other models for model fit in these cases, such as Comparative Fit Index (CFI), Normed Fit Index (NFI) and Non Normed Fit Index (NNFI), Goodness of Fit Index (GFI), and Adjusted Goodness of Fit Index (AGFI), where a value greater than or equal to 0.90 [42, 43] is considered to indicate good fit. Table 2 shows the other fit indices for the measurement model.

**Table 2:** Other structural model fit indicators

Fit indicators					
	**GFI**	**AGFI**	**CFI**	**NNFI**	**NFI**
Measurement model	0.94	0.91	0.97	0.96	0.95

As Table 2 shows, the fit indices indicate the desirable fit of the measurement model so that the markers can be used in the structural model.

### Ethical approval

Ethical approval was received from the institutional review boards of Lorestan University. All participants gave informed consent before participating in the study. This study was conducted in accordance with relevant guidelines and regulations.

Participants were provided with an informed consent form assuring them of anonymity, confidentiality, and the exclusively scientific purpose of the study. Participants were assured that their responses would be kept confidential and that their participation would in no way interfere with their studies. Those who agreed to participate in the study completed the questionnaire and returned it to the authors upon completion. Data in this study were also collected in person.

### Procedure

A questionnaire and an invitation letter that included parental consent and explained the purpose of the study were sent by the researchers to all schools in the last days before data collection and the schools sent the invitation letter to parents by email. A questionnaire with demographic items, parental support, active coping, hope, and resilience was collected by the researchers from the pre-selected classes in October 2019. Participants took part in the study voluntarily and anonymously, and students agreed to participate by completing an informed consent form attached to the questionnaire. Their data were also kept strictly confidential and used only for this study.

### Data analysis

All statistical data analyses were performed using Statistical Package for Social Sciences (SPSS) version 22.0 software. Spearman correlation analysis was performed to examine the linear relationships between each variable. The hypotheses were tested by structural equation modelling using LISREL 20 software. To test the fit of the model to the collected data, model fit indices were examined, including the chi-square test Fit Index (CFI), Normed Fit Index (NFI) and Non Normed Fit Index (NNFI), Goodness of Fit Index (GFI), and Adjusted Goodness of Fit Index (AGFI). In all analyses, a two-sided p-value of < 0.05 was statistically significant.

## Result

### Participant characteristics

Demographic variables in this study included gender, field of study, Academic year, Father's job, Number of children, mother's job, mother's education, Father's education and age.

Overall, 358 (63.92%) participants were girl and 202 (36.07%) were boy. Besides, 161 (28.75%) participants were in the first year of high school, 148 (26.42%) students were in second year, 131 (23.39%) students were in third year. The highest percentage of participants was aged 17–16 years (71%).

### Descriptive findings

Table 3 shows the mean and standard deviation of the variables in the study.

**Table 3:** Mean and standard deviation of the variables in the model

Agents	**Mean**	**Standard deviation**
Parental support (1)	19.06	5.11
Coping (2)	18.21	4.53
Resilience (3)	18.55	4.05
Hope (4)	18.05	3.93

### Relationship between parental support, coping, resilience and hope

Before testing the structural model of the research, it is necessary to examine the correlation of the variables in the model. Table 4 shows the correlation between the research variables.

**Table 4:** Correlation coefficients between the variables in the model

Factors	(1)	(2)	(3)	(4)
Parental support (1)	1.00			
Coping (2)	0.55**	1.00		
Resilience (3)	0.30**	0.52**	1.00	
Hope (4)	0.28**	0.40**	0.27**	1.00

*P* < 0.01^**^, *P* < 0.05^*^

As Table 5 shows, there is the highest correlation between coping and parental support (r = 0.55) and the lowest correlation between hope and resilience (r = 0.27).

**Table 5:** Demographic variables

		**Frequency**			**Frequency**	
**Gender**	**Boy**	202	**Father's education**	**High school**	24	4.28
	**Girl**	358		**Diploma**	260	46.42
**Field of study**	**Human**	192		**Bachelor’s degree**	252	45.00
	**Math**	215		**Master's degree and above**	24	4.28
	**Experimental**	153	**Mother's education**	**High school**	31	5.53
**Academic year**	**First year**	161		**Diploma**	241	43.03
	**Second year**	148		**Bachelor’s degree**	230	41.07
	**Third year**	131		**Master's degree and above**	58	10.35
	**Forth year**	120	**Mother's job**	**Employee**	203	36.25
**Father's job**	**Employee**	209				
	**Non- employee**	318		**Non-employee**	**357**	**63.75**
	**Retired**	34				
**Number of children**	**1–2**	474	**Age**	**14–15**	**142**	**25.35**
				**16–17**	**399**	**71.25**
	**3–5**	86		**18**	19	03.39

**Fig. 2:**
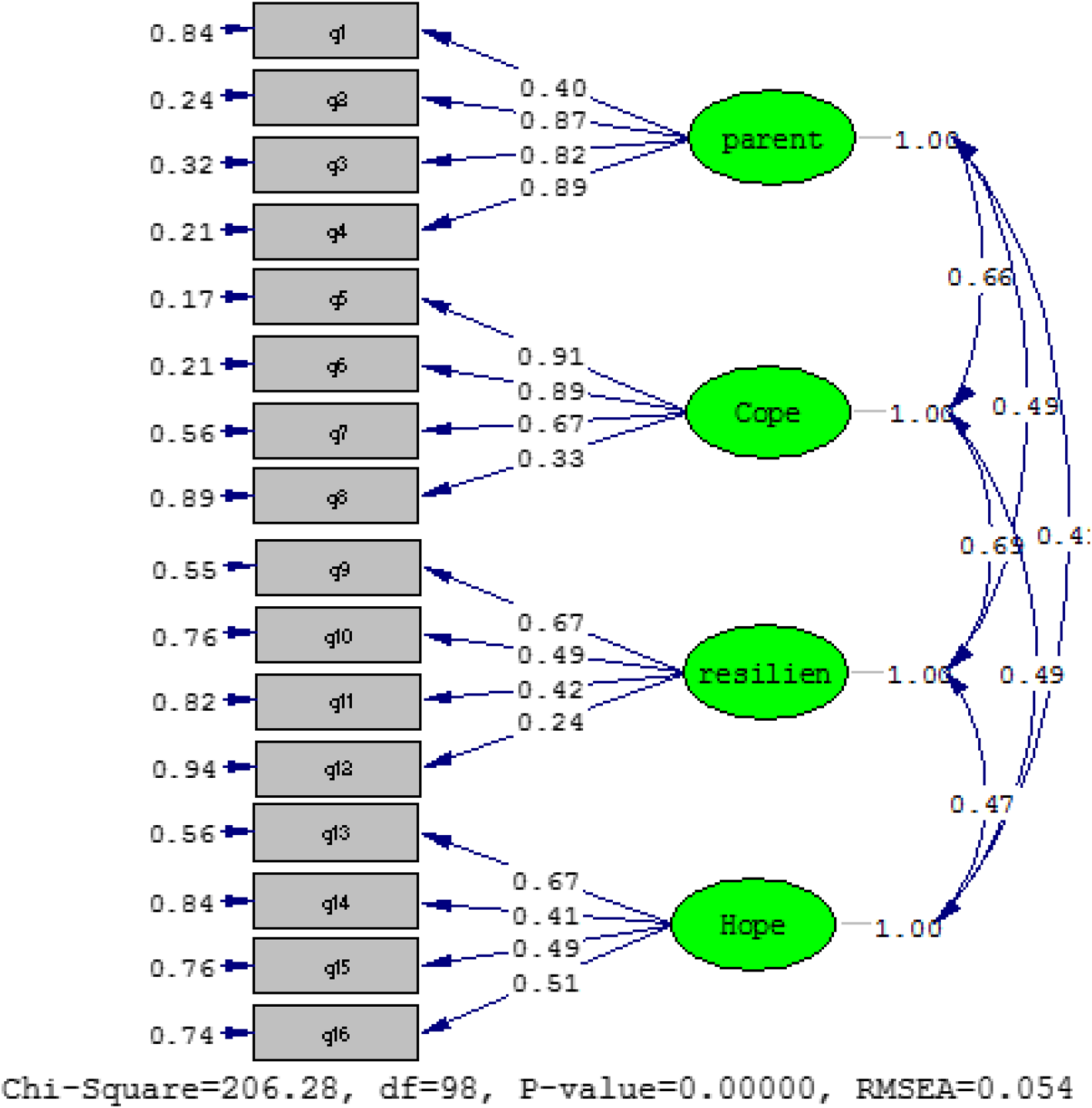
Standardized path coefficients, error value, chi-square index and root mean square error for the measurement model

The structural equation model was used to answer the two basic research questions about the effects of parental support on resilience and the mediating role of coping and hope. In the model, the structural equations of two models are tested. The first model includes the measurement model, whose indicators were presented, and the structural model, which expresses the relationship between the underlying variables. In the present study, LISREL software and the maximum likelihood method were used to fit the model. The results related to the implementation of the structural model, which shows the relationship between latent variables and some of the most important indicators for fitting the path analysis of the proposed model, are shown in Fig. 2.

**Fig. 3:**
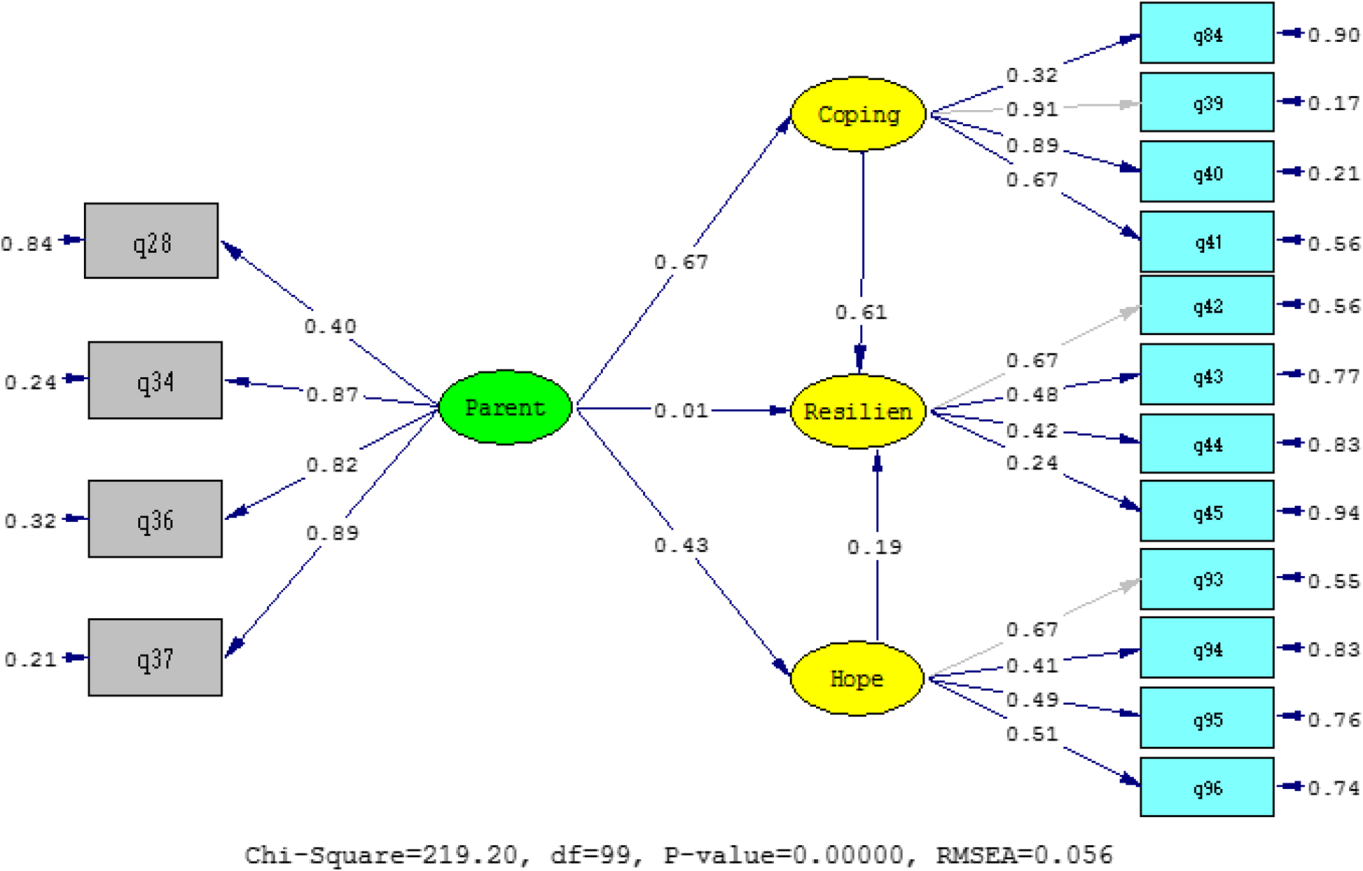
Standardized path coefficients, error value, chi-square index and root mean square error

As can be seen from Fig. 3, the conceptual model of the study shows a good fit, as the RMSEA index is in the desired range. Table 3 shows other fit indicators.

As Table 6 shows, the fit indices indicate the usefulness of the structural model. Table 3 shows the standardized direct and indirect path coefficients and the t-statistics.

**Table 6:** Other structural model fit indicators

Fit indicators					
	**GFI**	**AGFI**	**CFI**	**NNFI**	**NFI**
Structural model	0.93	0.91	0.97	0.96	0.97

As Table 7 shows, path coefficients are significant at the 0.01 alpha levels. The results of Table 8 show that parental support has a direct effect on coping (t = 10.9, β = 0.67), hope (t = 5.93, β = 0.43), and resilience (t = 0.09, β = 0.01). Coping also has a direct effect on resilience (β = 0.61, t = 6.44). Hope also has a direct effect on resilience (β = 0.19, t = 2.27).

**Table 7:** Coefficients and significance of indirect effects of model variables

Predictive variable	Variable criteria	Type of effect	βStandardized	t
Parental support	Resilience	indirect (total) by hope and active coping	0.49	5.98

*P* < 0.01^**^, *P* < 0.05^*^

**Table 8:** Coefficients and significance of direct effects of model variables

Predictive variable	Variable criteria	Type of effect	βStandardized	T
Parental support	Coping	Direct (total)	0.67	10.90
	Hope	Direct (total)	0.43	5.93
	Resilience	Direct (total)	0.01	0.09
Coping	Resilience	Direct (total)	0.61	6.44
Hope	Resilience	Direct (total)	0.19	2.27

*P* < 0.01^**^, *P* < 0.05^*^

As Table 7 shows, path coefficients are significant at the 0.01 alpha levels. The direct effects of the research model are shown in Table 7. The results of this table show that parental support for resilience is confirmed by the mediation of hope and active coping (t = 5.98, β = 0.49), so 0.49 of the changes in resilience are indirectly predicted.

## Discussion

The purpose of this study was to examine the structural model of resilience based on parental support: the mediating role of hope and active coping. The results show that the conceptual model of the study is consistent with the data. It can be concluded that there is a significant relationship between these variables. Individuals with high resilience have optimistic attitudes, positive emotions, and are open to new experiences. These emotions and positive attitudes lead to constructive behaviors and attitudes. Resilience helps individuals cope with uncertainty, conflict, and failure, and they are receptive to significant changes in life [44]. ​Resilience has received considerable attention in many disciplines over the past decade, including the social sciences, psychology, and family sciences [45, 46].

The first hypothesis of the present study suggests that parental support has an impact on resilience. based on self-determination theory (SDT), parents help meet adolescent's basic psychological needs, including autonomy, or the free choice of actions and desires; competence, or a sense of mastery over actions and events; and connectedness, or building a relationship with and supporting important people in their lives, which provides the foundation for them to achieve high levels of well-being and health [47].

Similarly, a child who is supported by peers or has had a similar loss experience may be motivated to express his or her feelings, which facilitates the normal grieving process [48]. Given young children's dependence on their parents and environmental conditions, family and community resources can have an important impact on adolescent's ability to adapt to adversity [28].

Parents facilitate their children's positive adjustment to stressful and threatening conditions through behavioral and emotional support. Parent who positively responded to their children by being kind, controlling, accepting, and supportive exhibited higher levels of resilience compared to children who did not have these experiences [49]. Research has also shown that perceived emotional warmth is positively related to resilience. Furthermore, perceived emotional warmth is a significant predictor of resilience [50, 51].

The present study confirmed the mediating role of coping and hope in the relationship between parental support and resilience. These findings are consistent with Shorey, Snyder, Yang & Lewin [52] and Satici [53] and contradict the research of Masten & Palmer [4] and Bornstein [54].

According to the control-oriented model of coping, active coping may involve various skills (such as problem solving and emotional expression) that can lead to behavioral and emotional adjustment in adolescents [21]. This adaptation may have an impact on resilience, as resilience is the ability to face and adapt to a difficult situation without being harmed. This finding is consistent with research by Compas et al. [13], McRae & Mauss [14], and Zimmer-Gembeck & Skinner [55]. Youth who receive parental support are more likely to try different solutions based on the support they receive [56]. These adolescents tend to be more receptive to advice and more creative. These characteristics may influence coping strategy, as indicated by the results of the present study [56]. This finding is consistent with the research findings of Shahrbabaki, Nouhi, Kazemi, & Ahmadi [57]. Even among adolescents who have high resilience and problem-solving, coping, and control skills, resilience is an important factor in coping with the demands of daily life.

Hope can influence resilience in ways that change how people evaluate stress, so that people with more hope face stressors and challenges in life positively. They also use more problem-solving strategies and are significantly less passive [58]. The results of the present study show that hope can have a significant impact on resilience. This finding is consistent with Snyder's [12] theory of hope, as well as the research findings of Bailey & Snyder [26], Horton & Wallander [27], and Lloyd & Hastings [59].

Parental support generally plays an important role in helping adolescents cope with challenges or problems they face. It can be concluded that parental support can influence hope because adolescents have institutionalized knowledge that they can cope in many cases regardless of the nature of the problem [60]. This finding is consistent with research by Kasler, Dahan & Elias [61].

Consistent with Snyder's [12] view, a supportive environment is associated with hope. Roswarski & Dunn [62] believe that people who face problems and believe that they are supported by the people around them, especially their parents, have more confidence to solve the problem and that the future will be better for them. Shorey et al. [52] reported that individuals with high hope had a strong emotional bond with at least one parent in their childhood. These parents were very helpful in gathering information about the goal and the attitude needed to achieve the goal, and they were a source of motivation and inspiration. In addition, people with high ambitions could rely on their parents and friends to help them deal with stress in critical situations. Consequently, parental support is an effective way to strengthen resilience by increasing self-confidence.

Grolnick et al. [63] assume that people have a need to feel connected to their parents and secure in their relationships with them. It follows that fathers and mothers who are responsive to their children's needs and exhibit warm, friendly, and encouraging behavior toward them satisfy their need for belonging by creating psychological safety. It seems that the warm and sincere relationship of parents with their children, which together with their feelings is unconditional, by creating a sense of belonging, connectedness and psychological security, is the basis for creating a positive attitude towards oneself, towards being. Engaging in new experiences and building warm, satisfying and intimate relationships with others gives them a sense of vitality, life force and hope to overcome difficulties. In contrast a negative environment (e.g., parental rejection) promotes perceptions of a short life expectancy and expectations that resources are unpredictable and unavailable [64, 65]. Parental rejection is a predictor of a wide range of behavior problems, including conduct disorder, externalizing behavior, delinquency, and substance use, indicating low resilience [66]. The parents' unsympathetic, angry, and dismissive attitudes and behaviors can affect the child's self-esteem and autonomy [67] and increase feelings of worthlessness and depression [67, 68]. All of these psychological functions are important antecedents of resilience reduction [29, 69, 70].

## Conclusion

The present study has several limitations regarding the generalizability of the results and the representativeness of the sample. The most important limitation was that this study contained a small sample, which could result in low power, greater risk of II type errors, and limited ability to detect and examine existing relationships and small effect sizes among other limitations, there were also limitations regarding the age of the participants. The majority of participants were older than 16 years, so this study was not able to examine a large sample of children in early to middle childhood. This study also did not examine the role of adolescents who live with one parent and are influenced by the support of a parent. Therefore, data on separated parents were not included in this study. Another limitation was the lack of research background, which theoretically limited the current study. Also only children self-report were used to assess parenting; parent’s perspective on their parenting may be different. Future research should explicitly take a multiple informant in the field of parents and children.

Examining parental support in a clinical sample could complement this research, as more variation in parental support may lead to more or different outcomes related to hope, resilience, and coping. Research has shown promising approaches to using the parental and family environment as a tool to prevent or treat depression in adolescents [71]. It is suggested that future studies further examine the factors that contribute to resilience in traumatized individuals, as well as parental separation and a broader age range.

Longitudinal studies covering the entire period of childhood and adolescence could also improve our understanding of differences in parental support and effects on hope, resilience, and coping. But, this study did not consider different ethnicities and regions. Future research is needed to examine this model in other regions.

Almost all of the studies examined were based on correlational data, so the results do not allow causal conclusions to be drawn. In the absence of further experimental data, it is not possible to determine whether parental support preceded children's resilience or occurred in response to it. Experimental studies should be aimed at improving coping strategies and resilience skills. Therefore, this study highlights prevention and intervention programs for the effects of parental support on hope, resilience, and coping.

Despite these limitations, this study contributes to the literature by further empirically validating known parental support and improving our understanding of the role of parental support in hope, resilience and coping, this conceptual framework has taken important steps in shifting from a pathological perspective to resilience. The present sample closely matched the population distribution in terms of educational level and father's occupation. Therefore, the present results may be generalizable to minority groups or families.

## Data Availability

The datasets used and/or analyzed during the current study are available from the corresponding author upon reasonable request.
